# Nanoplastics, Liver Injury, and Oxidative Mechanisms: Translating Animal Models Into Human Risk Assessment

**DOI:** 10.7759/cureus.86911

**Published:** 2025-06-28

**Authors:** Ruben Ruiz-Ramos, Mario A Luna-Palacios, Armando Pimentel-Toledano, Ana Laura Calderón-Garcidueñas, Julio C Viñas-Dozal, Francisco Ruíz-García, Noé López-Amador

**Affiliations:** 1 Faculty of Medicine, University of Veracruz, Veracruz, MEX; 2 Neuropathology, Instituto Nacional de Neurología y Neurocirugía, Mexico City, MEX; 3 Forensic Pathology, Institute of Forensic Medicine, University of Veracruz, Veracruz, MEX

**Keywords:** hepatotoxicity, liver injury, microplastics, nanoplastics, oxidative stress

## Abstract

Micro- and nanoplastics (MNPs) have emerged as environmental contaminants of increasing concern due to their potential to disrupt biological systems. The liver, as a key organ in detoxification and metabolism, is particularly susceptible to MNP-induced injury. This scoping review aimed to synthesize experimental evidence from preclinical animal models to identify the principal hepatotoxic mechanisms induced by MNP exposure and evaluate the implications for human health. A systematic search was conducted in PubMed, Scopus, Web of Science, and OpenAlex databases. Eligible studies included original research assessing hepatic outcomes in animal models exposed to MNPs. Data were charted for polymer type, particle size, exposure route, exposure duration, and toxicological endpoints. The review followed PRISMA-ScR guidelines and included 34 studies. Consistent hepatotoxic signatures were identified, including oxidative stress, inflammation, lipid dysregulation, and apoptosis. Polystyrene, polyethylene, and polylactic acid were the most studied polymers, with exposure via oral gavage or aquatic immersion. Mitochondrial dysfunction and genotoxic markers were also reported. The mechanistic convergence suggests shared injury pathways across particle types and models. MNPs induce liver injury through oxidative, inflammatory, and metabolic mechanisms, supporting their classification as emerging hepatotoxicants. While translational gaps persist, these findings highlight urgent research needs and inform environmental health risk frameworks.

## Introduction and background

The toxicity of micro- and nanoplastics (MNPs) has emerged as a critical concern in toxicology, particularly liver toxicity, with animal models revealing complex mechanisms of hepatic injury. Ingested MNPs can translocate from the gut to the liver, where they accumulate and trigger oxidative stress and inflammatory signaling via reactive oxygen species, compromising cellular integrity [1,2]. Furthermore, MNPs can act as vectors for hepatotoxins such as microcystin-LR, amplifying their toxic potential [3], while polymers like polylactic acid may degrade into more bioavailable oligomers, exacerbating hepatic accumulation and damage [4]. Commonly studied polymers now include polystyrene, polyethylene, polypropylene, polylactic acid, and polyvinyl chloride, reflecting their pervasive environmental presence.

Beyond cytotoxic effects, MNP exposure may alter gene expression and epigenetic regulation, potentially promoting chronic liver disease and carcinogenesis [5-7]. These findings align with epidemiological signals linking MNP exposure to metabolic liver disorders and malignancy [1,8]. Mitochondrial and lysosomal disruption has also been implicated, reinforcing the role of impaired organellar function in sustained liver injury [9]. Despite robust preclinical evidence, research gaps persist, particularly concerning chronic low-dose exposures and dose-response dynamics [2,9,10].

This scoping review aims to synthesize the experimental literature on MNP-induced hepatotoxicity in animal models, delineate mechanistic pathways, and assess implications for human health, thereby informing future hypothesis-driven research and regulatory frameworks. Specifically, we address the following PCC-framed question: in animal models (population), how does exposure to micro- and nano plastics (concept), including polystyrene, polyethylene, polypropylene, polylactic acid, and PVC, under controlled laboratory conditions (context), alter hepatic structure and function (outcome).

## Review

Methods

Protocol and Registration

This scoping review was designed a priori and registered on the Open Science Framework [54]. The protocol has remained unchanged throughout the review process.

Eligibility Criteria

We included in vivo animal studies that (i) exposed test subjects to micro- or nanoplastics of any polymer (polystyrene, polyethylene, polypropylene, polylactic acid, or PVC) by any route, (ii) reported at least one direct hepatic outcome (histology, serum enzymes, oxidative-stress markers, or gene-expression changes), and (iii) used a non-exposed control group. Conference abstracts, in vitro studies, and publications not reporting primary data were excluded.

Information Sources and Search Strategy

MEDLINE (via PubMed), Scopus, and Web of Science were searched from inception to 30 April 2024. The complete search strings and Boolean operators are available in the OSF registered protocol. No language or date limits were applied. Reference lists of eligible articles were hand-searched to identify additional studies.

Study Selection and Data Extraction

Title/abstract screening and the full-text review were performed independently by two reviewers. Disagreements were resolved by consensus. A predefined extraction template captured animal species, polymer type, particle size, exposure route, dose, duration, hepatic outcomes, and key mechanistic findings.

Data Synthesis

Findings were mapped descriptively and summarized narratively. A quantitative meta-analysis was prespecified to be conducted only if three or more studies reported an identical outcome using homogeneous metrics. No data set met this criterion; therefore, results are presented qualitatively.

Risk-of-Bias Considerations

Given the marked heterogeneity in animal models, exposure protocols, and outcome measures, applying a formal risk-of-bias tool (e.g., SYRCLE) would have yielded little interpretive value. Consequently, we focused on transparently reporting methodological features and limitations of each study within the narrative synthesis rather than generating an aggregate risk score.

Reporting Framework

The review adheres to the Preferred Reporting Items for Systematic Reviews and Meta-Analyses extension for Scoping Reviews (PRISMA-ScR) checklist. No amendments to the protocol were required.

Results

Selection of Sources of Evidence

**Figure 1:**
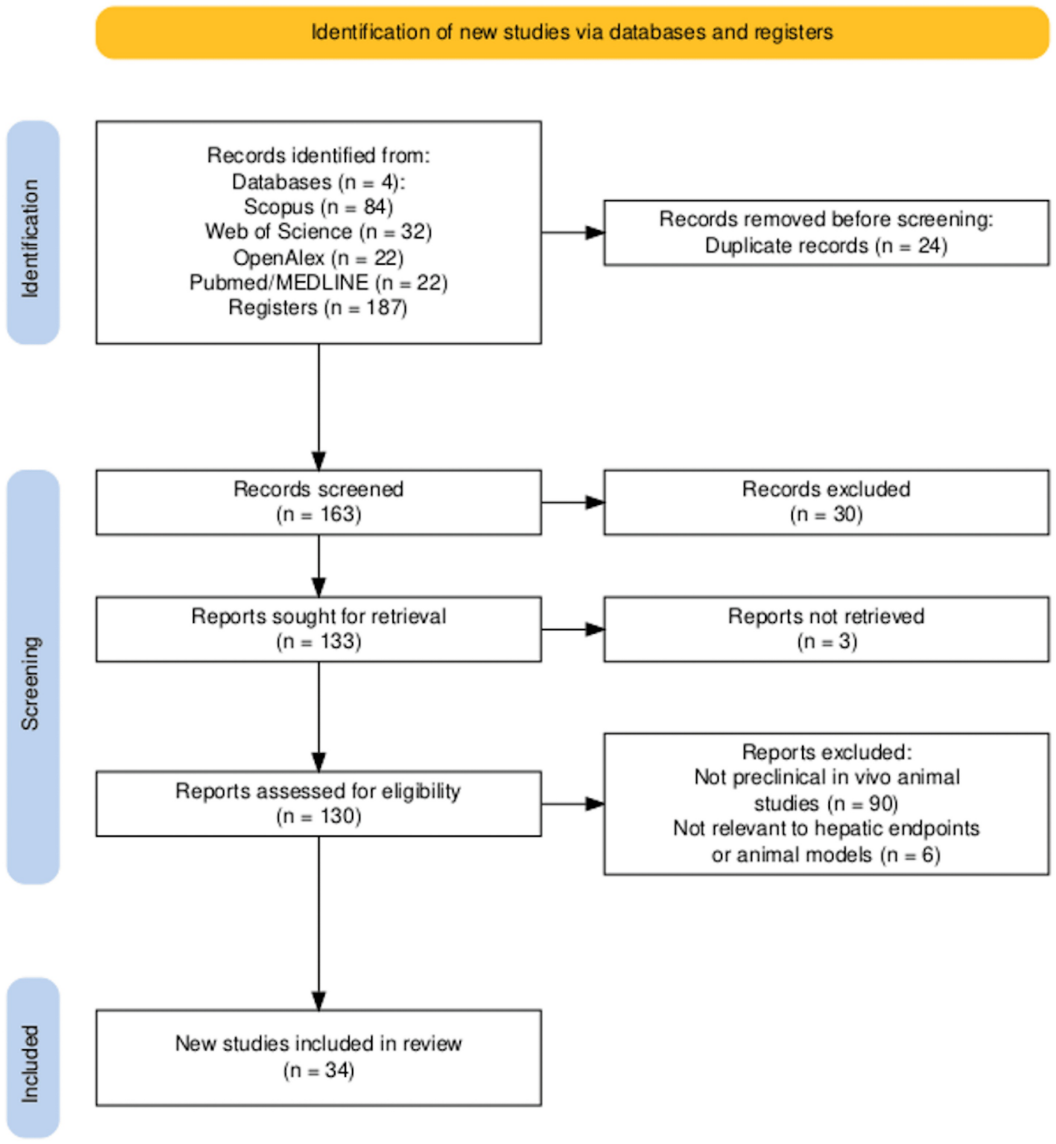
PRISMA 2020 flow diagram Process of identification, screening, eligibility assessment, and inclusion of studies in the scoping review on hepatotoxic effects of micro- and nanoplastics in animal models. The flow diagram was generated using the PRISMA 2020 Shiny App developed by Haddaway et al. (2022) [11].

A total of 187 records were identified through database searches: Scopus (84), Web of Science (32), OpenAlex (27), and PubMed (22). After removing 24 duplicates, 163 records remained. Title and abstract screening excluded 30 records (lack of abstract/DOI, reviews, or retractions). During the full-text screening, six articles were irrelevant to liver or animal endpoints, and 90 did not fulfill the in vivo criterion. Of the 37 full texts assessed, three were inaccessible, leaving 34 studies for synthesis. The selection process is shown in Figure 1.

General Characteristics

The 34 studies (2018-2024) employed rodents (rats, mice) and zebrafish most often; other models were rare. Exposure routes included oral gavage, aquatic immersion, and occasional intraperitoneal injection. Particle sizes ranged from nanoplastics (<100 nm) to microplastics (<5 mm) in spherical, fibrous, or fragmentary forms. Polystyrene was most common, followed by polyethylene, polylactic acid, and polyvinyl chloride (PVC); eight studies did not specify the polymer type. Exposure durations spanned 7-90 days, with doses from nanograms to milligrams kg⁻¹ day⁻¹ (or µg L⁻¹). Complete characteristics are in Supplementary Table 1 at the OSF HOME website [53]; a condensed summary appears in Table 1.

**Table 1:** Simplified summary of hepatotoxic mechanisms induced by micro- and nanoplastics across animal models This table provides a condensed synthesis of key findings from the included studies, organized by animal model, polymer, exposure route, and principal hepatotoxic mechanisms. It facilitates comparison by summarizing liver outcomes and citing supporting studies by author and year. The full dataset—including polymer, dose, exposure route, duration, and key findings—is available in Full Supplementary Table 1 at OSF HOME’s website [53].

Animal Model	Hepatotoxicity Mechanism & Outcomes	Studies Evidence (Author, Year)
Zebrafish (Danio rerio)	Oxidative Stress; ROS-Mediated Apoptosis/Inflammation; Lipid Disruption; Genotoxicity/DNA Damage; Endocrine Disruption; Xenobiotic/CYP450-Modulated Effects; ALT/AST elevation; Histopathological changes.	Xiong G, 2024 [33]; Ling, 2021 [43]; Wang L, 2024 [26]; Li Y, 2022 [37]; Rehman A, 2024 [34]; Luo T, 2021 [16]; Lu Y, 2016 [12]; Du J, 2024 [21]; Zhao Y, 2020 [!3]; Xie P, 2025 [20]; Şenol O, 2023 [19]; He J, 2020 [15]; Wen J, 2024 [31]; Wen J, 2025 [32].
			Mice (Mus musculus)	Oxidative Stress & Inflammatory Responses; Apoptotic/Cell Death & Mitochondrial Dysfunction; Reductive Stress; Lysosomal Damage; Fibrosis & METs-Related Mechanisms; Ferroptosis; Gut–Liver Axis Dysregulation; Kupffer Cell Pyroptosis; Microfiber-Induced Inflammatory Response; Apoptotic/Cell Death & Mitochondrial Dysfunction; ALT/AST elevation; Fibrotic changes; Insulin resistance; .	Wen S, 2022 [18]; Lee SH, 2024 [42]; Wen Y, 2024 [28]; Liang Y, 2024 [30]; Huang J, 2023 [41]; Shen Q, 2024 [29]; Wang Q, 2022 [25]; Wang S, 2023 [23]; Zhong G, 2022 [36]; Cheng W, 2024 [31]; Lu YY, 2024 [22]; Wang S, 2023 [27]; Ge Y, 2024 [40]; Wang L, 2023 [26]; Shi C, 2022 [17]; Qian X, 2024 [38]; Baek SM, 2023 [43].
						Rat (Rattus norvegicus)	Inflammation & Lipid Metabolism Disruption; ALT/AST elevation; Histopathological changes.	Ma Y, 2024 [41].
						Whiteleg Shrimp (Litopenaeus vannamei)	Oxidative Stress; ALT/AST elevation.	Yu Y, 2023 [32].

Critical Appraisal

In keeping with the scoping review methodology, no formal risk-of-bias tool was applied. Qualitative inspection noted frequent omissions in particle morphology, exposure duration, or control-group detail; these limitations are highlighted in the discussion.

Results of Individual Sources of Evidence

Thirty-four experimental studies converge on a pattern of MNP-induced liver injury across models. Early work by Lu et al. [12] showed hepatic accumulation of 70 nm and 5 µm polystyrene particles in Danio rerio, provoking oxidative stress, structural damage, and metabolic disruption. Zhao et al. [13] confirmed metabolic dysregulation after 21 days of PS-MP exposure, while Xiaodong et al. [14] demonstrated that PS-NPs enhanced microcystin-LR toxicity via oxidative stress. Structural damage without overt biochemical change was documented by He et al. [15]; Luo et al. [16] reported synergistic toxicity when PS-MPs were combined with imidacloprid.

Mammalian studies echoed these findings. Shi et al. [17] described gut-liver-axis disruption and lipid deposition in mice, and Wen et al. [18] showed microbiota-mediated sensitization to cyclophosphamide. Thermal stress amplified PS-NP hepatonecrosis in zebrafish (Senol et al. [19]), and aging increased vulnerability (Xie et al. [20]). Du et al. [21] and Lu et al. [22] linked PS-NPs with hepatocyte hypertrophy and lysosomal dysfunction. Rodent work by Wang et al. [23-27] revealed size-dependent necroptosis or apoptosis via PTEN/PI3K/AKT/autophagy signaling and MET-driven fibrosis. NRF2-NLRP3 suppression was highlighted by Wen et al. [28].

Potential countermeasures were explored: selenium nanoparticles (Shen et al. [29]) and maltol (Liang et al. [30]) mitigated PS-NP injury by restoring mitochondrial dynamics and autophagy. Aged polypropylene MPs produced stronger effects than pristine ones in mice and human liver organoids (Cheng et al. [31]). Vector phenomena persisted in aquatic models: PS-MPs enhanced HBCD uptake in shrimp (Yu et al. [32]). Co-exposures (tire-derived particles, arsenic, and PCB-77) intensified steatosis, apoptosis, pyroptosis, and autophagy via NLRP3, PI3K/mTOR, and MAPK pathways [33-39]. Inhalation studies implicated ferroptosis (Ge et al. [40]); chronic exposure remodeled hepatic lipidomics (Ma et al. [41]). Size-dependent immunotoxicity was detailed by Huang et al. [42]. Ethanol-induced mucosal disruption increased systemic MP accumulation (Baek et al. [43]). Beyond polystyrene, polyethylene microfibers caused progressive degeneration (Razzaq et al. [44]), while sub-micron PS-MPs promoted NAFLD-like changes (Lee et al. [45]).

**Figure 2:**
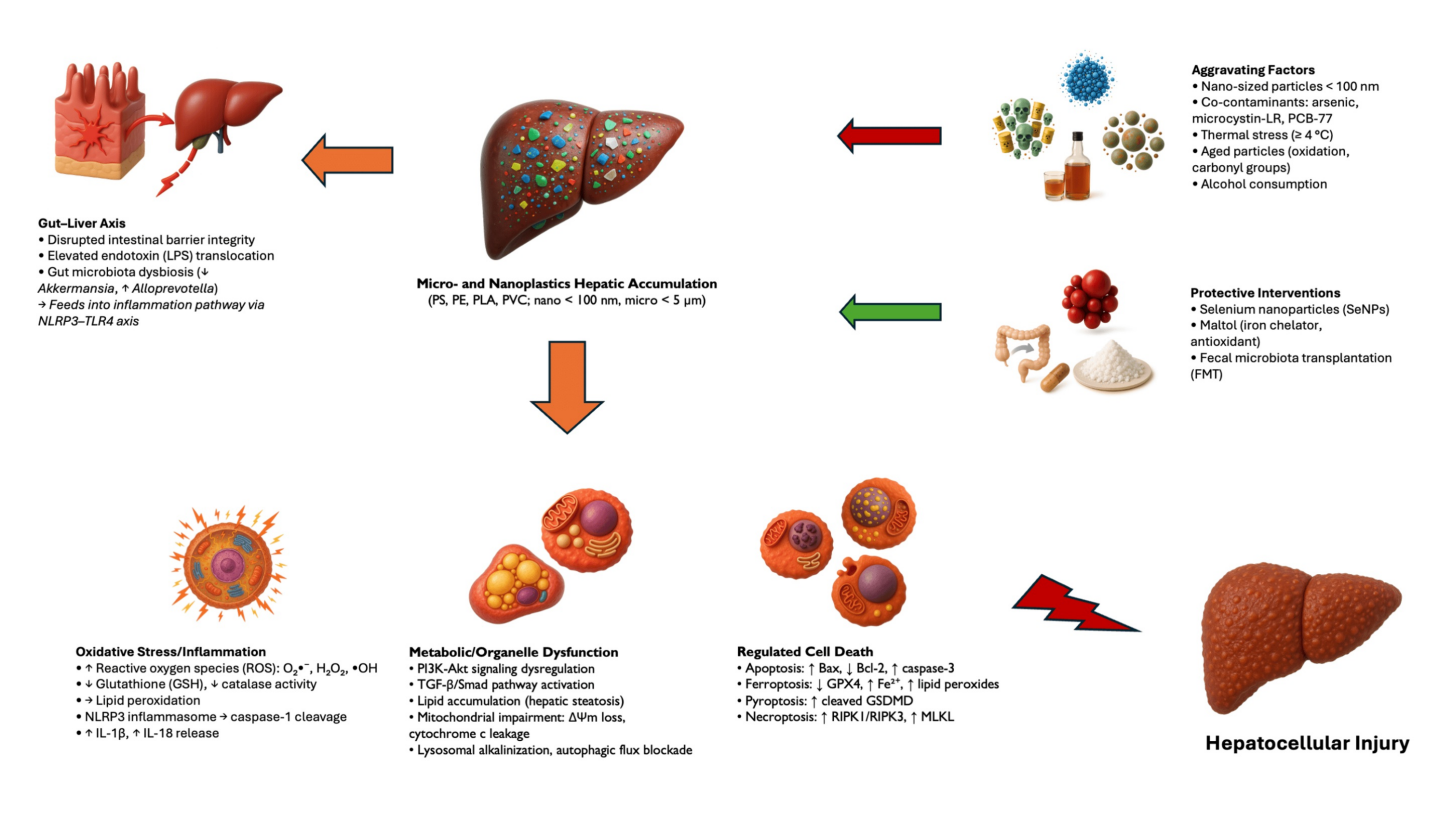
Mechanistic overview of micro- and nanoplastic-induced hepatotoxicity via oxidative, inflammatory, metabolic, and cell death pathways The image is created by the author.

The mechanistic convergence of oxidative stress, inflammation, and regulated cell death is summarized schematically in Figure 2.

Synthesis of Results

Across studies, MNP exposure elevated ALT/AST, induced hepatocyte vacuolation, necrosis, mitochondrial and lysosomal dysfunction, lipid dysregulation, and early fibrosis markers. Zebrafish and rodent models dominated, with polystyrene and polyethylene particles most examined. Collectively, these data support a biologically plausible link between MNP exposure and liver injury (Table 1).

Discussion

Summary of Evidence

In plain terms, the liver is especially susceptible to damage from tiny plastic particles that move from the gut into the bloodstream. A growing body of preclinical work shows that exposure to polystyrene micro- and nanoplastics (PS-MNPs) provokes reproducible hepatotoxic patterns in aquatic and mammalian models. Particle size and physicochemical traits dictate biodistribution: nanoplastics (<100 nm) penetrate systemic circulation more readily than larger microplastics, accumulating in hepatic tissue [35,46]. Chronic or high-dose exposure to microplastics (<5 µm) also results in liver deposition [45].

Across zebrafish, mice, and rats, typical histological lesions include inflammatory infiltration, vacuolar degeneration, lipid accumulation, and early fibrosis, closely mirroring non-alcoholic fatty liver disease (NAFLD) [16,18,45]. Oxidative stress is a central driver: excess reactive oxygen species (ROS), diminished glutathione and catalase, and elevated malondialdehyde compromise antioxidant defenses [23,30]. Multiple regulated-cell-death programs contribute-apoptosis (Bax↑, cytochrome c↑, Bcl-2↓) [31,39], pyroptosis via NLRP3/caspase-1 in Kupffer cells [37], as well as ferroptosis (GPX4↓) and necroptosis (RIPK3↑) [47].

PS-MNPs further disrupt hepatic metabolism through PI3K/Akt and TGF-β/Smad signaling, elevating serum AST and ALT [30,48] and compromise the gut-liver axis by weakening intestinal barriers and altering microbiota, thereby amplifying endotoxin-driven inflammation [16,38]. Importantly, sub-chronic models show partial reversibility once exposure ceases [19], suggesting threshold-dependent injury that nonetheless warrants caution over cumulative, lifelong exposure.

Interpretation and Implications

The recurring triad of oxidative stress, inflammation, and mitochondrial dysfunction parallels classic xenobiotic liver injury and underpins early NAFLD pathology. Although doses in animal studies exceed estimated human exposure, the same molecular motifs appear in in vitro and biomonitoring data [49,50]. These findings support including hepatic biomarkers in environmental risk assessments, especially for vulnerable groups such as children, pregnant individuals, and those with metabolic disease [51,52]. Under-reporting of particle attributes (shape, aging state, surface chemistry) limits ecological validity, yet the consistent injury pattern across polymers suggests that the oxidative-inflammatory milieu, rather than any single particle property, drives the pathogenicity, a hypothesis demanding targeted study.

Strengths and Limitations

This review offers one of the most comprehensive syntheses of MNP-induced hepatotoxicity to date, bridging mechanistic evidence across species, polymers, sizes, and exposure routes. The emphasis on pathways such as mitochondrial disruption, ferroptosis, and lipid dysregulation advances current understanding. Limitations include the absence of formal quality appraisal, high inter-study heterogeneity, and predominant reliance on high-dose, short-term exposures that may not mirror real-world conditions. The exclusion of non-English and grey literature could introduce publication bias. Incomplete reporting of particle surface properties further constrains reproducibility and external validity.

Future Directions

Priority should be given to longitudinal animal studies using environmentally realistic, chronic low-dose regimens with weathered particles, integrating multi-omics to identify early biomarkers. Incorporating sex as a biological variable and sensitive developmental windows will enhance translatability. Human cohort studies with rigorous exposure quantification (e.g., fecal or serum plastic burden) are essential to link MNPs to steatosis, enzyme elevation, and fibrosis. Nested case-control designs within liver disease registries could clarify the role of MNPs in disease progression. Advanced causal inference tools, Mendelian randomization, Bayesian networks, and machine learning, are needed to disentangle interactions with diet, alcohol, co-pollutants, and genetics. Finally, collaborative platforms must standardize particle characterization, exposure protocols, and regulatory thresholds to translate laboratory insights into public health policy.

## Conclusions

This scoping review provides compelling evidence that micro- and nanoplastics (MNPs) consistently induce hepatotoxic effects in animal models through convergent mechanisms involving oxidative stress, inflammation, metabolic disruption, and organelle dysfunction. Across diverse polymers, exposure scenarios, and species, the findings demonstrate a recurring pattern of hepatic injury with implications for non-alcoholic fatty liver disease and systemic inflammation. While methodological variability and high-dose exposures limit direct extrapolation to human health, the mechanistic coherence across studies reinforces the biological plausibility of MNP-induced liver toxicity. These insights underscore the urgency of advancing both experimental and epidemiological research to elucidate the long-term impacts of MNP exposure on hepatic and systemic health.
